# What Is the Impact of Accidentally Transporting Terrestrial Alien Species? A New Life Cycle Impact Assessment Model

**DOI:** 10.1021/acs.est.3c08500

**Published:** 2024-02-08

**Authors:** Jan Borgelt, Martin Dorber, Charly Géron, Koen J. J. Kuipers, Mark A. J. Huijbregts, Francesca Verones

**Affiliations:** † Industrial Ecology Programme, Department of Energy and Process Engineering, Norwegian University of Science and Technology (NTNU), Trondheim 7034, Norway; ‡ Biodiversity and Landscape, TERRA research centre, Gembloux Agro-Bio Tech, University of Liège, Gembloux 5030, Belgium; § Plants and Ecosystems, University of Antwerp, Wilrijk 2610, Belgium; ∥. CNRS, ECOBIO (Écosystèmes, Biodiversité, Évolution), UMR, University of Rennes, Rennes 6553, France; ⊥ Department of Environmental Science, Radboud Institute for Biological and Environmental Sciences, Radboud University, Nijmegen, GL 6500, Netherlands

**Keywords:** Life Cycle Impact Assessment, invasive species, characterization factors, supply chains, transport, ecosystem quality

## Abstract

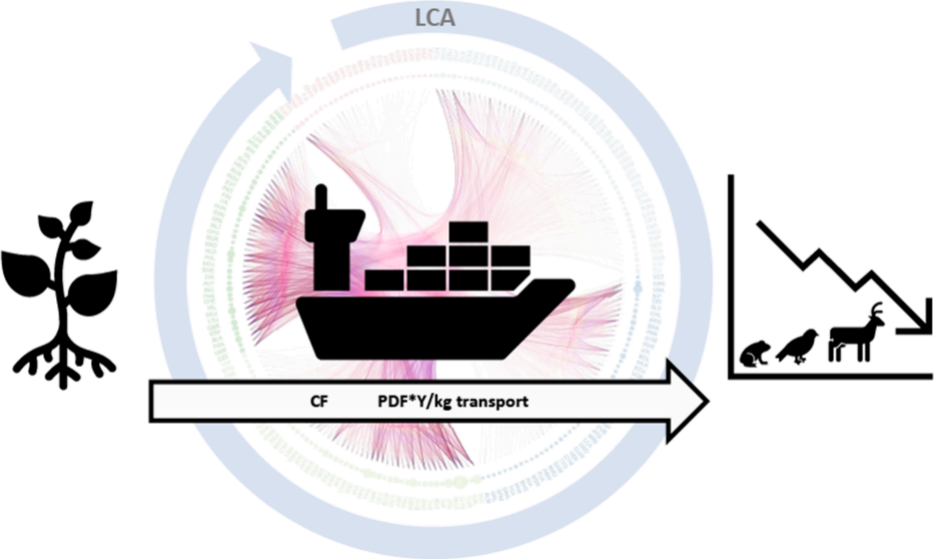

Alien species form one of the main threats to global biodiversity. Although Life Cycle Assessment attempts to holistically assess environmental impacts of products and services across value chains, ecological impacts of the introduction of alien species are so far not assessed in Life Cycle Impact Assessment. Here, we developed country-to-country-specific characterization factors, expressed as the time-integrated potentially disappeared fraction (PDF; regional and global) of native terrestrial species due to alien species introductions per unit of goods transported [kg] between two countries. The characterization factors were generated by analyzing global data on first records of alien species, native species distributions, and their threat status, as well as bilateral trade partnerships from 1870–2019. The resulting characterization factors vary over several orders of magnitude, indicating that impact greatly varies per transportation route and trading partner. We showcase the applicability and relevance of the characterization factors for transporting 1 metric ton of freight to France from China, South Africa, and Madagascar. The results suggest that the introduction of alien species can be more damaging for terrestrial biodiversity as climate change impacts during the international transport of commodities.

## Abstract 2

Although detrimental, the impact assessment of transporting alien species on terrestrial biodiversity is not yet operationalized within Life Cycle Assessment. This study quantifies the potentially disappeared fraction of native terrestrial species due to the introduction of alien species per unit of goods transported between two countries.

## 1 Introduction

Globalization has spurred a notable upturn in international trade. Within these expansive global trade and transportation networks, unintentionally contaminated goods or transport vessels stand out as one of the primary catalysts for the introduction of alien species.^1−8^ The type of species introduced includes plants, fungi, viruses, and bacteria, but also animal species of various sizes, from arthropods to fish, reptiles, amphibians, and mammals.^9,10^ These alien species, defined as species appearing outside of their natural range due to human agency,^11−14^ are observed across all continents,^15^ and the rate at which they colonize new areas continues to rise.^16−18^ This has been especially the case starting from the second half of the 20th century.^16,18−20^

Anthropogenic activities, as well as environmental conditions of the recipient system, crucially influence the rate of arrival and establishment success of alien species.^21,22^ Alien species that manage to reproduce independently over large areas and/or cause substantial negative impacts are named “invasive alien species”.^23^ Invasive alien species can have devastating impacts on ecosystem dynamics in multiple ways, even with implications for human health^24−26^ and the economy.^27,28^ Their ecosystem impacts range from altering environmental conditions and disrupting food webs^29^ to triggering declines in species diversity.^30−34^ These invasive alien species are one of the biggest threats to global biodiversity.^23,35^

Our understanding of the invasion processes has improved substantially in recent decades. Extensive analyses have been conducted aiming to identify relevant drivers of introductions^21,36^ and its relative contributions,^37^ as well as more detailed approaches that predict the risk of invasion across regions,^38,39^ species,^40^ and among trade exchange.^41−45^ In addition, besides early detection and management of alien species in the early stages of their invasion process, proactive prevention of alien species introductions has been found to be the cheapest and most effective way for minimizing their consequences on invaded ecosystems.^38,46−51^ Yet, avoiding introductions of alien species in a globalized and interconnected world is an intricate challenge.

One possibility for reducing the consequential damage of alien species is to promote less detrimental activities in the technosphere.^52^ Life Cycle Assessment (LCA) is a tool for quantifying environmental impacts in a comparative manner throughout entire value chains while considering resource uses, transportation flows, and emissions.^53^ In LCA, it is possible to assess the combined impacts of several pressures simultaneously to highlight hotspots of impacts and mitigation options, making it an effective decision-support tool.^53^ LCA consists of several phases, including the goal and scope definition, life cycle inventory (collection of resource uses and emissions), and the life cycle impact assessment (LCIA). In the latter phase, characterization factors (CFs) are used to quantify environmental impacts of specific human activities and impact pathways. A lack of CFs for a certain type of pressure or impact means that this impact cannot be considered. As of today, many impacts of the main anthropogenic biodiversity threats can be assessed in LCA, for example, land use,^54^ pollution (ecotoxicity),^55^ or climate change.^56,57^ However, an LCIA methodology to quantify impacts of invasive alien species is yet to be developed.^58−60^ So far, only one LCIA case study for the introduction of alien species into freshwater systems through inland shipping in the Rhine and Danube Rivers is published.^61^ This in turn means that although transportation is a fundamental part of any attempt aiming to holistically assess the environmental consequences within LCA of, for instance, a product, impacts caused by invasive alien species cannot be accounted for. Hence, current LCAs looking at trade^62^ may underestimate the overall environmental consequences of transporting commodities, which can lead to misleading conclusions in decision making.

To address this research gap, we developed country-to-country-specific CFs with global coverage to quantify the damage of introduced alien species to terrestrial ecosystems caused by the transportation of goods by building on the work of Hanafiah et al.^61^ The CF quantifies the ecosystem damage per kilogram of transported goods in the importing country using the metric of the potentially disappeared fraction (PDF) of terrestrial species. We modeled the fate of alien species introductions by analyzing data on bilateral transportation flows from 1870 to 2019,^63−65^ estimated species relocations across 255 countries,^16,18^ and impacts of alien species on native terrestrial species based on data from the IUCN Red List of Threatened Species.^66^

We demonstrate the applicability of our CFs in a case study by comparing the impacts of transporting freight to France from China, South Africa, and Madagascar. In parallel, this showcases how the inclusion of alien species impacts can change the interpretation and conclusion of an LCA.

## 2 Materials and Methods

### 2.1 Overview of the Characterization Factor

We developed CFs for biodiversity impacts related to the spread of alien species via the transportation of goods. The regional CF describes the ecosystem damage per kg of transported goods in the importing country (i) from an exporting country (e) as the PDF of native species [PDF × year × kg^–1^] (eq 1). The CFs were obtained by multiplying country-to-country-specific fate factors (FF) that describe alien species introductions (in this study, based on proxy data from vascular plant species) per kg of transported commodity, with a constant effect factor (EF) that quantifies the impact on species in invaded ecosystems (in this study, amphibians, birds, and mammals). It is important to note that we do not distinguish between different stages of invasion but assume that an increase in alien species is monotonically related to an increase in invasive alien species and its adherent impacts.
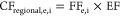
1

The quantified impact represents the regional relative species losses. Impacts relevant at a global scale were estimated by combining regional impacts and global extinction probabilities (GEP).^67^ The GEP estimates the consequences of regional species loss for global extinctions. We multiplied the regional CFs by country-specific GEPs that were averaged across all impacted species groups to convert them to global CFs (eq 2).
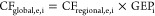
2

### 2.2 Fate Factor

#### 2.2.1 Approach

The FF describes the change in the alien species fraction (ASF) integrated across year and transported quantity [ASF × year × kg^–1^]. Thereby, the FF for the transportation route from exporting country e to importing country i FF_e,i_ was estimated by quantifying the change in the number of alien species in relation to the total number of species in the importing country i (ΔASF_i_) caused by the average yearly amount of transported commodities in that time period (ΔTR_e,i_) (eq 3).
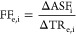
3

We consider the spread of alien species via the transportation of traded commodities from exporting nations e into importing nations i. The global commodity transportation network thereby acts like a dispersal vector and facilitates unintentional introductions of alien species that ultimately lead to a change in the fraction of alien species in the importing country. The ASF was calculated for country i as the fraction of extant alien species (AS) in relation to all species present, native (*S*), as well as alien (eq 4).
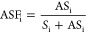
4

#### 2.2.2 Quantities of Commodity Transportation

Data on quantities of bilateral trade (i.e., transportation) flows in metric tons were retrieved from BACI, an international trade database at the product level.^63^ BACI is based on official international trade statistics provided by the Commodities Trade Statistics database of the United Nations (https://comtrade.un.org/). We selected the longest available period of the data set (1995–2019, version 2021–02) and converted the data from metric tons into kg. In addition, we obtained trade data from the Correlates of War project (Version 4.0)^64,65^ that reports on monetary trade values exchanged between countries from 1870 to 2014. We used linear regression to extend the BACI timeseries and convert monetary trade exchange from the Correlates of War project into transported quantities [kg]. For each combination of importing and exporting country, one regression model was fitted using first- and second-order polynomial terms of the trade flow and a fixed intercept at zero. The average *R*^2^ of the predicted data was of 0.83 (Figure S1). We henceforth calculated the cumulative transported quantity in 10 year timesteps for each combination of exporting and importing country, giving us estimates on the cumulative exchanged quantities for 15 timesteps, that is, from 1879 to 2019.

The nearest distance between importer and exporter was calculated based on a global administrative borders shapefile and the R package *sf.*(68)

#### 2.2.3 Alien Species Data

Data on the alien species presence was retrieved from the alien first records database (version 2),^16,18^ including information on the approximate year of introduction and the invasion status for 26,052 individual species across 276 different regions of the world. Our core objective in the FF calculation is to quantify the link between the transported quantity and alien species introductions. We therefore considered species irrespective of the invasion status. Subnational regions were matched to countries whenever necessary to align with the resolution of the trade data sets.

Because the availability and accuracy of data vastly differ across different species groups, we consider vascular plants as proxy organisms for unintentional species introductions. Vascular plant species have been shown to be relevant for the unintentional transportation alongside commodities in previous studies^19,69,70^ and make up the largest and most comprehensive share of the consolidated data set (*n* = 14,013). Hence, in this study, we quantify the number of accidentally transported vascular plant species due to the transportation of goods and assume this being representative of unintentionally transported species.

**Figure 1:**
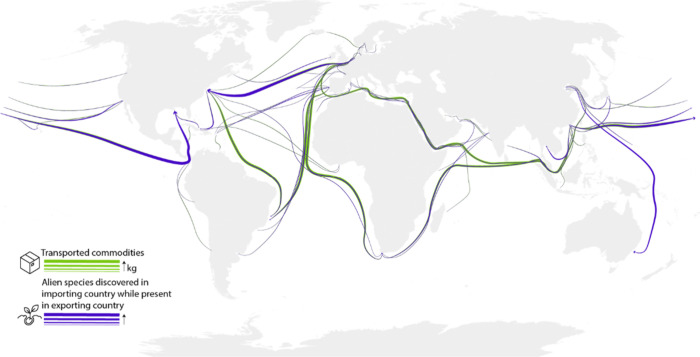
Illustration of the gathered data set including transportation flows of traded commodities in kilograms (green) and estimated alien species transportation, that is, alien species that were discovered in the importing country while present in the exporting country (purple).

We retrieved native countries of occurrence for 47,675 terrestrial vascular plant species from Borgelt et al.^71^ For species that were not covered by that source, but are included in the alien first record database, we retrieved a list of native countries of occurrence from plantsoftheworldonline.org using the R packages *rvest* and *taxize*. We then counted the number of native species within each country. We further defined the species pool for each country in each year in the period 1870 to 2019, that is, the number of species being available for being introduced into other countries. In each year, the species pool thereby consists of all native species and all species being already introduced by that year, to account for bridgehead effects, that is, the fact that species may further be transported outside their native distribution.^72,73^ We then estimated the number of alien species being transported each year and for each combination of importer and exporter. We assumed that this number approximates the number of alien species being recorded for the first time in a given year in importing country i, while belonging to the species pool of exporting country e (Figure 1). Finally, we aggregated the number of transported species to 10 year time intervals to reduce uncertainty in the exact timing of the first records, between 1879 and 2019.

For each combination of importing and exporting country, the following information was available in the collected data set: the cumulative transported quantity (TR_e→i_), the nearest distance (*D*_e→i_) between both countries, the number of species present in the exporting country (*S*_e_) and the number of native species in the importing country (*S*_i_) as independent variables, and the estimated number of species relocations between both countries (AS_e→i_) as response variable. TR_e→i_, *S*_e_, and AS_e→i_ were measured in 10 year time intervals, while *D*_e→i_ and *S*_i_ are constant.

#### 2.2.4 Calculation Procedure

We quantified the effect of commodity transport (TR) on alien species introductions via a generalized linear mixed model (GLMM) using the R package *lme4*(74) in the statistical software R version 4.0.3^75^ in Rstudio version 1.4.1103.^76^ The GLMM was used to estimate the relationship between the number of introduced alien species (AS_e→i_; independent variable) and the amount of transported commodities (TR_e→i_), transport distance (*D*_e→i_), number of species in the exporting country (*S*_e_), and number of native species in the importing country (*S*_i_) as explanatory variables (or moderators/fixed effects) while accounting for variability caused by uncontrolled factors (i.e., random effects). Because the number of alien species transported between country pairs represents count data and follows a Poisson distribution, we fitted the GLMM with a negative binomial distribution and logarithmic link function to avoid overdispersion.^77^ TR_e→i_, *S*_i_, and *S*_e_ were log transformed to stabilize variance and reduce spread of the data and to consider a nonlinear relationship between the response and independent variables. We expect nonindependence in the data set due to varying effect sizes depending on unmeasured factors in the importing and exporting countries (e.g., due to proactive invasion control measures), as well as different environmental conditions within donor and recipient regions. This was controlled by including the importer and the exporter nested within each time step as random effects:^77^ (1|period/country_i_) + (1|period/country_e_). We fitted multiple models to consider all possible combinations of fixed effects, with the most complex model being:
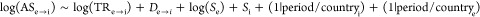
and simpler models containing subsets of these explanatory variables. The best supported model was selected based on Akaike’s Information Criterion (AICc) (Table S1). Since correlated variables could affect model estimates,^78^ we tested for collinearity using the Spearman rank correlation coefficient. All correlations between pairs of variables were lower than ±0.5, indicating that no highly correlated variables were included in our analyses.^77,78^

We then calculated marginal and average FFs with the fitted GLMM. The difference between marginal compared to average FF is the calculation of the number of transported alien species. For average FFs, the GLMM was used to calculate the expected number of alien species in 1870 (i.e., at TR = 0 kg) and in 2019 (i.e., the total cumulative transported quantity) for each trade partnership. To obtain country-to-country-specific FFs, the resulting change in number of alien species was divided by the total cumulative transported quantity exchanged between both countries [kg] over the entire time period (i.e., 150 years, from 1870 to 2019) and weighed according to the number of species present in the importing country (eq 2).

The marginal FF was calculated similarly except that the change in alien species was calculated as the difference in alien species transported between 2019 and one additional year of transportation (i.e., the annually transported quantity averaged across the past 10 years). Henceforth, the resulting change in alien species was divided by the additional TR (kg) over time (i.e., 1 year) and weighed according to the total number of species present in the importing country.

Since, we expect higher uncertainty for low reported trade quantities, and we did not calculate FFs for combinations of countries with an exchanged cumulative quantity of less than 100 kg per year.

### 2.3 Effect Factor

#### 2.3.1 Approach

The calculation approach for the EF was adapted from Hanafiah et al.^61^ for use in terrestrial ecosystems. The consequential change in the present alien species affects ecosystems negatively in the importing country. The EF describes the potentially disappeared fraction of native terrestrial species due to a change in the alien species fraction [PDF × ASF^–1^] (eq 5). We do not distinguish between different stages of invasion for the alien species considered as the unit of the EF must directly relate to the unit of the FF.
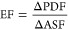
5

#### 2.3.2 Alien Species Fraction

The number of native alien vascular plant species in each country was counted as in the FF approach, that is, from Borgelt et al.^71^ and from plantsoftheworldonline.org. Alien species counts of vascular plants per country were retrieved by summing all of the first records from a given country from the alien first records database. The ASF was then calculated for each country as the fraction of alien species in relation to all species present (eq 4).

#### 2.3.3 Potentially Disappeared Fraction of Species

The potentially disappeared fraction of species (PDF) is the metric for impacts on “ecosystem quality” that is currently recommended by the life cycle initiative hosted by UN Environment.^79^ For the sake of consistency and for being comparable to other impact categories in LCA,^61,80^ we quantify the consequential impacts of alien species introductions in terms of a regional PDF of native terrestrial species. In each country, we listed all native terrestrial species based on data from the IUCN Red List of Threatened Species and retrieved threats as well as Red List categories for each species using the R package *rredlist*.^81^ We only consider species groups with comprehensive information about their conservation status and threats assessed by the IUCN (i.e., amphibians, birds, and mammals). In this study, the PDF represents the regional fraction of species that already went extinct or is currently threatened by extinction, suggesting that if the relevant threats are not diminished, those species disappear eventually.^61^ Hence, the PDF in country i is the fraction of species in the Red List categories vulnerable (VU), endangered (EN), critically endangered (CR), and extinct/extinct in the wild (EX), which experience “Slow, Significant Declines”, “Rapid Declines”, or “Very Rapid Declines” due to “invasive non-native/alien species” according to the IUCN Threat Classification Scheme,^66^ in relation to the total number of native species (*S*_i_) in country i (eq 6). In total, 6% of the species in our data set (*n* = 32) were labeled as data deficient (DD) at the IUCN Red List. This label does not represent an extinction risk category but indicates a lack of the required ecological knowledge to perform a Red List assessment. Therefore, we considered three different options for calculating the PDF: (1) we only included those DD species that were previously predicted to be threatened by extinction,^82^ (2) we considered all DD species as being not threatened by extinction, and (3) we considered all DD species as being threatened by extinction.
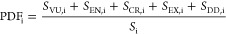
6

However, because the assessment detail, as well as the number of available IUCN assessments, likely differs across countries,^83^ we consider *S*_i_ the number of native terrestrial species for which both threats and threat severity have been assessed and are not stated as “unknown” at the IUCN, that is, only species whose threats have been assessed at a comparable level of detail.

#### 2.3.4 Calculation Procedure

Because species can be threatened by other confounding factors, we used quantile regression to establish a stressor–response relationship, following the approach of Hanafiah et al.^61^ Least-squares regression techniques aim to fit a relationship between explanatory and response variables through the mean. In quantile regression, the response can represent any part of its probability distribution.^84^ Quantile regression is especially powerful for filtering out confounding factors in noisy explanatory data that obscure the true response^84^ and has been successfully applied to link exposure–response curves, for example, for pollution.^85−87^ If the response is limited by confounding factors, quantile regression based on one of the upper boundaries of its probability distribution (e.g., the 95th percentile) is expected to reflect the response’s relationship to the corresponding explanatory variable.^84^ Conversely, one of the lower boundaries (e.g., the 5th percentile) reflects a relevant relationship if confounding factors boost the response. We expect the estimated PDF to be primarily increased by other additional threats to a species. Therefore, we fitted a quantile regression through the fifth percentile of the data using the R package *quantreg.*(88) However, since the fitted slope is largely affected by the chosen percentile, we fitted curves through the 5th, 10th, and 15th percentile to explore potential effects on the outcome of this study. Since we assume lower uncertainty related to data from larger countries, the magnitude of country size, that is, the decimal logarithm of the area [km^2^], was used as the weighting factor within the quantile regression. Only countries with data in all used data sets (IUCN, plantsoftheworldonline.org, and alien first records database) were considered. The slope of the quantile regression then represents the EF, describing the expected change in regional PDF per unit change in ASF as a constant (eq 5).

### 2.4 Application Example

We assess the transport of 1 metric ton of freight by container ship from (i) China, (ii) South Africa, and (iii) Madagascar, to France to evaluate the relevance of considering impacts of alien species introductions relative to other pressures affecting biodiversity. The generated CFs and existing climate change CFs were applied to assess the effects of transporting 1 metric ton of freight between the respective countries and to compare the corresponding global PDFs. The countries were chosen based on transportation history and distances between the trading partners. We retrieved inventory data on emissions of a standard container ship from ecoinvent version 3.8,^89^ shipping distances between trading partners from the SeaRates database (https://www.searates.com/services/distances-time/), and global average CFs for climate change in terrestrial ecosystems from LC-IMPACT.^90^

## 3 Results

### 3.1 Fate Factor

The marginal and average FFs indicate that transported freight respectively causes a median change of 4.6 × 10^–14^ and 1.2 × 10^–10^ ASF × year per kg, with a high variability across countries (i.e., interquartile range of ±1.3 × 10^–12^ and 3.2 × 10^–9^ ASF × year/kg, respectively). The GLMM utilized for calculating FFs draws on the continuous variable transported quantity, the size of the species pool in the exporting country, and the geographic distance between trading partners as well as number of native species in the importing country. All retrieved parameter estimates were significant (*p* < 0.001) (Table S2) and indicate positive relationships of alien species records to the scaled log level of trade quantity (9.3 × 10^–3^ ± 5.3 × 10^–4^), native diversity (3.3 × 10^–1^ ± 6.3 × 10^–2^), as well as species pool of the exporting country (8.4 × 10^–1^ ± 1.2 × 10^–2^), and a negative relationship of distance between exporter and importing country (−3.6 × 10^–5^ ± 6.6 × 10^–7^). The marginal and conditional *R*^2^ values calculated following Nakagawa et al.^91,92^ suggest that 7% of the total variability is explained by the fixed effects only and 99% is explained by both fixed and random effects (Table S2). However, the GLMM shows increasing variance of the residuals with increasing fitted values, that is, heteroscedasticity, and an unbalanced spread across fitted values toward lower values (Figure S2).

### 3.2 Effect Factor

**Figure 2:**
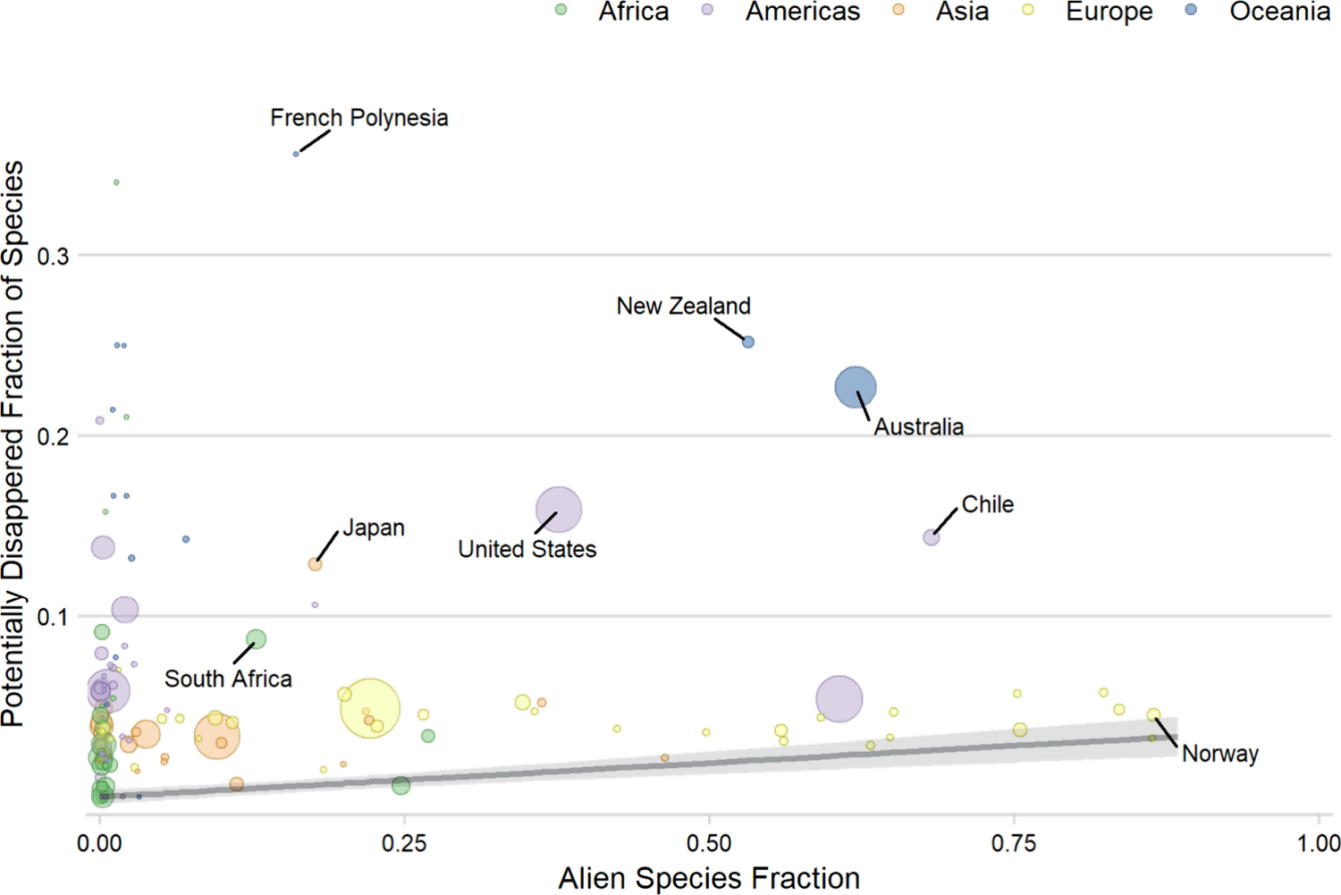
Estimated relationship between alien species fraction (ASF) and potentially disappeared fraction of species (PDF) based on country-level data (*n* = 140). The size of the bubbles corresponds to relative differences in the country area. The effect factor (ΔPDF/ΔASF) was estimated by fitting a quantile regression through the 5th percentile (gray line) because species may be threatened by several factors at the same time. The 95% confidence interval of the quantile regression is shown in light-gray.

The EF describes the expected change in the regional PDF per unit change in ASF as a constant and is given by the slope of the quantile regression through the fifth percentile, that is, 0.038 (lower: 0, upper: 0.046) PDF/ASF (Figure 2). The corresponding slopes through the 10th and 15th percentile were 0.038 PDF/ASF (lower: 0.032, upper: 0.048) and 0.028 PDF/ASF (lower: 0.017, upper: 0.043), respectively. The slope through the 5th percentile did not differ when all data deficient species were considered not threatened or threatened.

### 3.3 Characterization Factors

**Figure 3:**
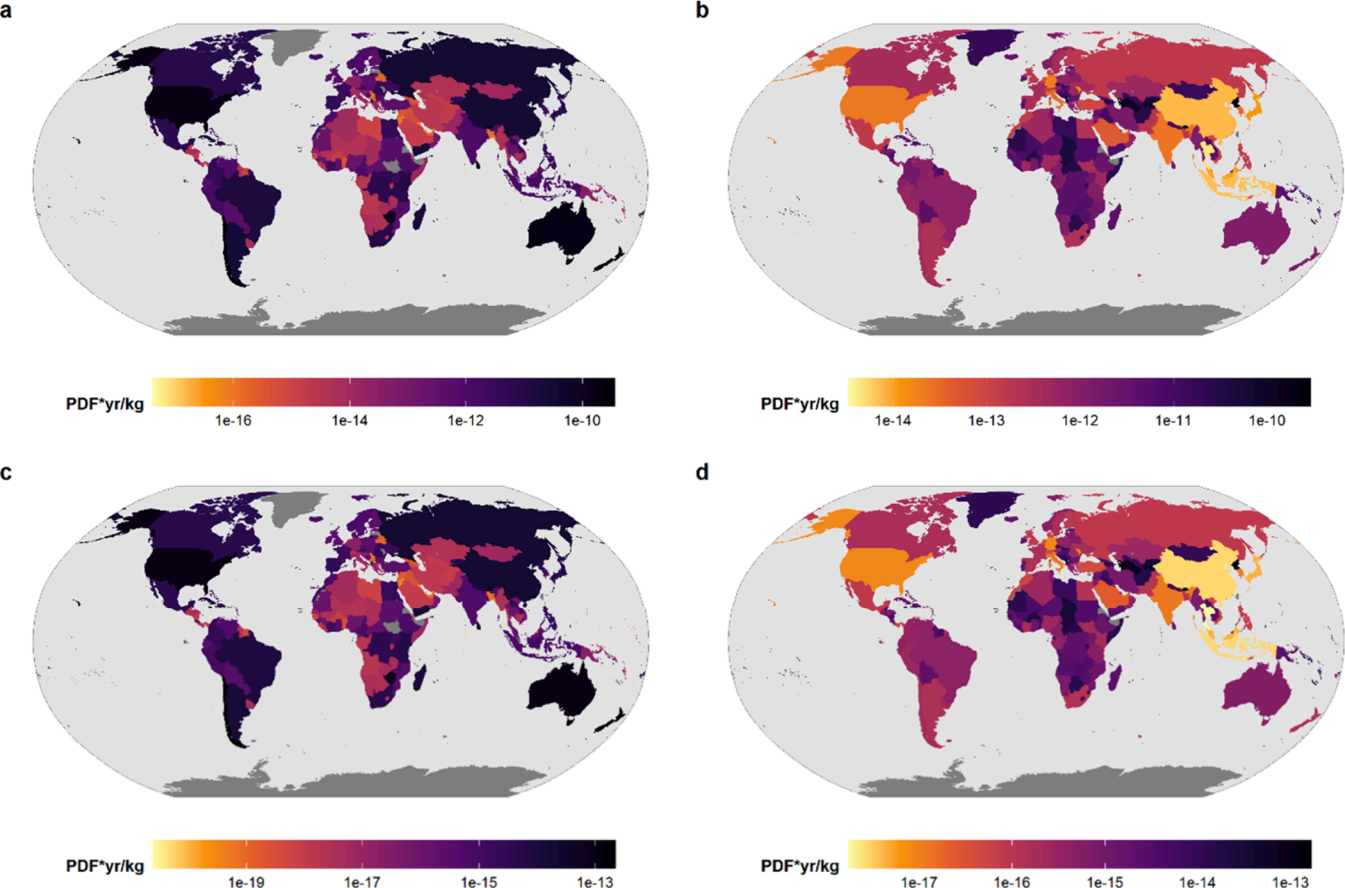
(a, b) Mean average and (c, d) marginal global characterization factors (CF) for each importing country (left panel) and for each exporting country (right panel). The CF describes the impact in the potentially disappeared fraction (PDF) × years per kg of transported goods. The left panel illustrates impacts experienced within each importing country, while the right panel indicates mean impacts caused by the respective exporting countries.

CFs for 33,259 combinations of countries were generated, including all countries with active trade partnerships. The marginal and average global CFs indicate highly variable impacts across bilateral trading partners with a median of 1.4 × 10^–18^ and interquartile range of ±4.3 × 10^–17^ and 3.6 × 10^–15^ ± 1.0 × 10^–13^ PDF × year/kg, respectively. The marginal and average CFs are highly correlated suggesting that the ranking among importing and exporting countries remains constant (Figure S3). The mean impact per imported kg was highest in New Zealand, Chile, and Australia. This was the case for both marginal and average CF. In contrast, the lowest impacts per imported kilogram occurred in countries with low global extinction probabilities, such as Nauru and Bahrain or highly industrialized countries such as Belgium and several countries in central Europe (Figure 3). The lowest global average CFs were obtained for combinations of particularly high trade, that is, often neighboring countries such as United Arab Emirates to Bahrain (2.6 × 10^–23^ PDF × year/kg), distant countries, for example, Sweden to Tuvalu in the Pacific Ocean (2.4 × 10^–20^ PDF × year/kg), or low global extinction probabilities in the recipient country, for example, imports to Bahrain from Brazil (8.0 × 10^–23^ PDF × year/kg).

### 3.4 Application Example

**Figure 4:**
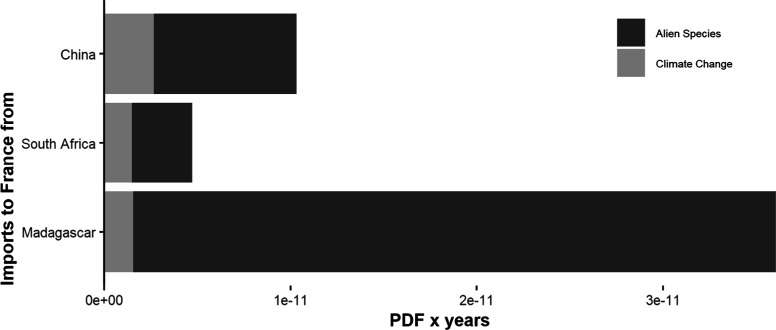
Potentially disappeared fraction of species (PDF) × years in terrestrial ecosystems for 1 metric ton transported between different countries caused by climate change and alien species introductions. Characterization factors for climate change were retrieved from LC impact.^90^

The application example suggests that alien species can contribute a considerable part to the impacts caused by the transportation of goods. Across the trading routes taken as examples, impacts from transported alien species could be more severe than climate change impacts (Figure 4). In addition, while transporting freight from both South Africa (distance approximately 12,100 km) and Madagascar (distance approximately 12,609 km) to France has very similar environmental performances in terms of climate change, our CF for average impacts on PDF indicates significantly lower overall impacts for goods transported from South Africa to France compared to Madagascar to France. Similarly, in terms of climate change impacts, freight transport from Madagascar to France would be favorable compared to exports from China to France (transported distance of approximately 21,549 km). However, once alien species impacts are accounted for, the overall environmental performance is different. We want to stress that, while, for example, climate change impacts scale linearly with both increasing distance and transported quantity, our CFs indicate impacts from alien species irrespective of distance as it is implicitly included in the FF calculations. Thereby, alien species impacts tend to be less relevant compared to, for example, climate change effects, in freight that is being transported over larger distances, because of the negative relationship of distance to relocated alien species (Table S2) caused by, presumably, decreasing survival chances when transported over large distances.

## 4 Discussion

### 4.1 Fate Factor

Marginal and average FFs span over more than 10 orders of magnitude indicating that global extinction risk due to species invasion varies greatly with trade partner.^41^ In terms of absolute predicted number of alien species introductions during one additional year of trade (i.e., in the marginal approach), large exporting nations,^27^ as well as some megadiverse countries,^2^ represent the greatest potential sources of alien species for the rest of the world, including China and the USA. Because the marginal FF considers alien species that possibly occur due to 1 year of additional trade, the highest number of alien species per kg is expected to be transported between countries with relatively low bilateral trade exchange in the past. In contrast, the number of additional alien species per kilogram of freight transported between frequent traders is expected to be low. This supports earlier studies suggesting that the accumulation of alien species can be described using a variant of species–accumulation curves, that is, species–import curves.^41−43^ Hence, the initially transported goods are likely to account for the highest number of introduced alien species to the recipient region, often consisting of the most common species in the exporting region. Any additional transport of commodities is considered to cause fewer alien species introductions because the species pool of the exporting region gets exploited over time.^42^ While the marginal approach gives an indication about possible future impacts, the average FF offers a retrospective view of already transported freight and occurring alien species introductions. Both in the marginal and average approaches, alien species introductions are expected to be higher at relatively shorter distances between importing and exporting countries and if the exporting country has a larger species pool. In addition, the number of transported alien species is weighed by the number of species present, alien as well as native, in the importing country to obtain the change in ASF. Consequently, the FF reflects that an additional alien species has a smaller effect on this change in ASF in species rich countries and countries that already contain a high number of alien species.

Ideally, information about the exact introduction pathway of species would be utilized for studying a causal link between transportation flows and alien species introductions. However, such data is scarce and only available for a small subset of species and species groups.^93,94^

For instance, the accidental transportation and subsequent introduction of alien species into new environments are not typically a linear process. Instead, intermediate stops pose opportunities for some alien species to leave the transport vessel and for others to board, leaving the exact origin of introduced alien species often unknown. In addition, it is assumed that small as well as long-living propagules are likely candidates for accidental transportations,^6,43,69,95^ while some species can be transported intentionally.^96^ Without such data at hand, we used a GLMM to establish a link between the transportation history of bilateral trade partnerships and the accompanying unintentional alien species introductions. In these calculations, we treat each transportation route, including all stops, as an individual transportation flow. The analysis is consequently tailored to statistically estimate how trade, measured in kilograms transported, affects the number of alien species introduced to the destination country from various source countries. Thereby, we allow all possible transportation routes to contribute to an alien species’ introduction if the species is present in the exporter. Hence, if an alien species is present in two exporting countries, we account for the contribution of both countries to the potential invasion in the importing country in estimating the impact of transportation on the total number of introduced species.

The results of the GLMM suggest that there are additional factors influencing the number of introduced alien species between countries that were not included in the FF calculations (Table S2). A possible reason may be our focus on total bilateral transport quantities only, while ignoring the differentiation between type of commodity and type of transport vessel. In principle, for each type of transport (i.e., ship, airplane, truck, or railway), different pathways are responsible for the majority of unintentional alien species introductions, for example, stowaways on transported commodities or stowaways on or in the transport vessels itself.^5^ Thereby, the spread of invasive species depends on the type of commodity,^97^ their respective quantity or volume,^41−43,98^ as well as the overall connectivity between countries.^19,36,70^ Although accidental alien species introductions are more likely to occur via some specific commodities and types of transport,^5,19,69,97,99^ the detailed contribution by each individual pathway remains unknown.^19,97^ Future developments of the proposed approach may therefore focus on implementing additional explanatory variables, such as the separation between different transport modes, different commodities, and the overall connectivity between countries.

In fact, although evolving, the availability and quality of data on transportation flows and alien species introductions remain incomplete. We found bilateral exchanged quantities only available for a limited time period, that is, 1995–2019, which excluded a large part of the history of species introductions available in the alien first records database.^18^ Hence, we were faced with the dilemma of using comprehensive but short-term, transportation data in concert with a consequently limited subset of alien species introductions or estimating transported quantities in the past based on additional data sources. We decided to combine two widely used trade data sets^63,64^ for obtaining extrapolated values on transported quantities spanning more than a century back in time. Using linear regression for estimating transported quantities back in time introduces additional uncertainty in our results. However, we found that, in terms of transportation flows, the majority occurred after 1995 (i.e., 82% of the predicted quantities; Figure S4). We therefore do not assume major effects of the regression estimates on the outcome of the FF calculations, while allowing for the consideration of additional timesteps with known alien species introductions.

However, the trade and transportation network have evolved throughout the time horizon of our data set. Several technological developments contributed to the rise of alien species invasions, including faster transportation times, the use of shipping containers, and change in ballast.^16,100,101^ As a consequence, the main introduction pathways of alien species evolved simultaneously. For instance, faster transportation allowed more species to survive the journey^102^ and generally increased rates and frequencies of global trade.^95^ The use of freight containers further streamlined international trade, enhancing efficiency but also contributing to alien species introductions through, for example, the accumulation of debris.^100^ The transition from solid ballast, in the form of rocks or gravel, which could lead to the unintentional spread of terrestrial alien species, to the use of ballast water, shifted toward more aquatic organisms being transported.^101^ Hence, the relevancy of different introduction pathways likely differed between the start (1870) and end (2019) of the data set that we consolidated. Ideally, each individual pathway would be considered separately in the analysis. However, data that would allow for a more accurate representation of these factors in our calculations are currently not available. We therefore analyzed the data irrespective of exact introduction pathway but instead focused on the combined effect of all unintentional introduction pathways. This includes unintentional species introductions via ballast, cargo, shipping containers, as well as shipping vessels. By focusing on the overall effect, we aim to counteract the apparent historical differences in relevancy.

In addition, we decided to employ data from the alien first records database for studying patterns in alien species introductions. This source provides somewhat exact timings of arrivals for individual alien species, is especially for plant species one of the most comprehensive existing data sources, and is presumably less biased than more unstructured data sets, for example, from the Global Biodiversity Information Facility.^16,18^ However, we still expect differences in data quality when comparing different regions of the data set.^103−105^ Moreover, both naturalized^95^ and invasive species^106^ often only become apparent after a time lag. The number of alien species transported between countries was therefore restricted. On the one hand, by limiting environmental conditions in the recipient region that could restrict establishment^4,22,107−109^ and on the other hand, by incomplete data on species arrivals.^104,110,111^ While environmental conditions in the importing country are implicitly included in the fitted GLMM, incomplete occurrence data may lead to false absences of alien species in some regions. This could directly affect the estimated effect sizes of the GLMM. As a result, both marginal and average FFs can be considered conservative estimates and could be higher if the utilized alien species data set was complete.

Furthermore, the predicted number of transported alien species seems to be more uncertain at higher predicted values. However, the relevant regression coefficients for calculating the FF remain valid. That is, the number of transported alien species increases with the quantity of goods being transported (TR).

### 4.2 Effect factor

The EF was retrieved based on country-level data on the degree of invasion (i.e., ASF) and an estimated potentially disappeared fraction (PDF) of native amphibians, birds, and mammals. For estimating the EF, we followed a previous approach^61^ and used quantile regression because species can be threatened by more than one factor, causing potentially additive effects across different environmental threats. The slope of the quantile regression represents the EF and describes the expected change in the PDF per unit change in ASF. According to this slope, about 4% of the native species in a country will be lost if it will be dominated by alien species, that is, an EF of 0.038 regional PDF/ASF. We acknowledge that the retrieved slope is difficult to validate but argue that it is a pattern emerging from the data,^112^ linking the confirmed presence of alien species to their potential impacts on native species as monitored by the IUCN Red List. For establishing this relationship, we fitted a curve through one of the lower percentiles. Not all countries contributed equally to the quantile regression analysis, but instead, data obtained from larger countries were given a higher weight. This approach was chosen to counteract the effect of presumably incomplete data from smaller countries on the analysis. However, many data points, for example, Australia, New Zealand, and French Polynesia, were above the fitted curve through the 5th percentile (Figure 2). The effect of choosing the 10th or 15th percentile for calculating the EF was minor, but the lowest percentile (i.e., the 5th percentile) is expected to offer the most conservative estimate of overall effects of a change in ASF. In addition, we found that there were only few data deficient species (*n* = 32) in our analyses, representing 6% of the total number of considered species for calculating the EF. Hence, the inclusion or exclusion of data deficient species did not have major influences on the estimated EF. Therefore, we expect that the utilized predictions of whether these species are likely to be threatened or not^82^ to represent the most appropriate compromise.

The impacts of alien species on native ecosystems are of diverse nature.^113^ Even within the same taxonomic group, the impacts can be multifaceted, and different taxonomic groups could pose different risks to the invaded systems. The impacts of invasive vascular plants, for instance, can differ from those of other taxonomic groups in their ecological consequences. Invasive plants may affect native species and ecosystem processes by altering habitat structure and resource availability.^114^ In contrast, alien species of other taxonomic groups, for example, mammals, insects, or microorganisms, can exert direct effects on native species, through, for example, predation,^115^ or by triggering disease outbreaks^116^ with broader ecological implications. Although their impact varies, both invasive plant species, as well as alien species of other taxonomic groups, share the capacity to disrupt ecosystems and pose negative consequences across various species groups.^23,117,118^ However, limiting this study to a small subset of species was inevitable as data for additional taxonomic groups were not consistently available. We selected alien plant species as proxy for unintentional species introductions, based on data availability and reliability.^16^ In addition, we selected mammals, amphibians, and birds as comprehensively assessed groups being representative of impacted species in the invaded system. Invasive plants have been shown to impact native fauna in several ways,^117,118^ however, we want to stress that the impacts presented here do not necessarily represent a causal link between alien plant introductions and impacts on native mammals, amphibians, and birds but rather a correlation. The implementation of additional taxonomic groups may therefore increase the robustness of the proposed framework.

Furthermore, several other factors are likely to affect the response of a native community to species introductions, and not all introduced alien species become invasive. However, in this study, only native as well as alien species richness was considered to calculate the EF, irrespective of invasion status. Once more data on direct links between alien species and negatively affected species become available, the development of mechanistic impact pathways could improve the accuracy of future effect models. As such, future attempts may explore the utility of alternative approaches for quantifying the effect of alien species introductions, such as their effects on species–area relationships,^119^ considering relative impact potentials,^120^ or accounting for invasibility of ecosystems.^5^

### 4.3 Characterization Factor

In this study, we have proposed a methodology with global coverage to account for impacts of alien species introductions from the transportation of goods. Powerful and extensive databases, on, for example, trade data,^63,64^ origins and threats of species,^66^ and introduction dates of alien species,^16,18^ in concert with statistical models were vital for generating the CF of this study because detailed monitoring data of alien species are largely unavailable.^12,121^ This study presents a first attempt at the global implementation of impacts originating in human-mediated movements of alien species into the framework of LCIA.

The generated CF is applicable only for terrestrial species. The complexities and nuances of impacts and responses within different ecosystems, combined with variations in data availability and transport mechanisms, present distinct challenges in assessing invasive species’ impacts across terrestrial, freshwater, and marine realms. As a result, our study has chosen to concentrate solely on terrestrial ecosystems to ensure a focused and comprehensive analysis. While we acknowledge the importance of considering impacts on other ecosystems, we believe that assessing impacts on different realms separately aligns with the current standards of LCIA.^79^ This approach allows for a more detailed and tailored examination of the unique characteristics and implications within each ecosystem, which is essential for maintaining the accuracy and depth of our analysis. Hence, for marine and freshwater ecosystems, additional CFs need to be developed.

For better comparability across other impact categories and regions, we used global extinction probabilities (GEPs)^67^ to translate regional species losses to global extinctions. Even though both the EF and GEP are based on data from the IUCN, there is no double counting. The EF is quantified based on the number of species that are significantly threatened by invasive species, that is, excluding least concern and near threatened species. In turn, GEPs are calculated using spatial information and all threat levels of species irrespective of the exact reason for threat.

The LCA framework is able to provide a comprehensive view of a product’s life cycle and its environmental impacts, yet it necessitates the use of simplified and generalized models.^122^ Consequently, our approach is limited in fully capturing the intricacies of biodiversity impacts.^59,123^ This highlights the inherent challenge in reconciling the holistic perspective offered by LCA with the nuanced nature of ecosystems. For instance, biodiversity impacts in the LCA framework are currently assessed in terms of species loss only and include a time dimension, expressed in PDF × year.^79^ This implies that the impacts are reversible. Note, however, that for the case of alien species introductions, cost-intensive and active eradication are required for reversing the impact.^46,48^ We further stress that there are other impacts, which are not covered by the proposed CF. For example, studies have shown that alien species cause a multitude of impacts,^124^ ranging from changing habitats^29^ to indirectly benefiting native species.^125^ Although extinctions may occur,^30,34,126^ a variety of impacts, including the homogenization of biodiversity,^33,127^ can occur simultaneously. Hence, we advocate for exploring additional ways to account for the various impacts of alien species within the framework of LCA.

We want to emphasize that the generated CFs should primarily be used in hotspot analyses, for example, for identifying countries with relatively high or low trade-related invasive species impacts. When conducting a cross-comparison with other impact categories, it is important to consider the discussed uncertainties and limitations. Additionally, we highlight that the developed CFs are not intended to replace local environmental impact assessment studies.

The presented approach contributes to existing efforts to develop a more comprehensive list of impacts covered in LCA, aiming toward more holistic impact assessments. The provided CFs are relevant for various efforts that aim to assess transportation impacts within global supply chains. The results of our case study suggest, in line with previous attempts,^61^ that impacts from alien species can contribute considerably to overall impacts caused by the transportation of goods. Until now, LCA-based decision making could not consider this specific impact pathway and thereby necessarily underestimated the total possible environmental consequences, with the cost of species relocations into new environments effectively being considered as an externality.^128^ In turn, being able to incorporate the impacts of accidental transportation of alien species into the framework of the LCA facilitates the identification of mitigation options. Effectively, this may help not only to reduce the overall environmental impact^23^ but also to mitigate the substantial costs^49^ associated with alien species introductions resulting from the global transportation network.

## Data Availability

All gathered data and R code for analyzing the data is available in a GitHub repository (https://github.com/jannebor/cf_ias_transport).

## Supporting Information Available

Additional tables and figures related to results and statistical analyses (PDF)
The CFs, provided as Excel spreadsheet, are organized into separate sheets for both the marginal and average approaches; within these approaches, CFs are detailed for each country-to-country combination, focusing on those with an average traded quantity of at least 100 kg per year; CFs for combinations with lower quantities are not provided; the “importer” and “exporter” are identified by their three-letter ISO codes; the impact of regional transport on biodiversity is given in the “CFregional” column, which measures the potentially disappeared fraction of species (PDF)*year per kilogram transported between the specified countries; similarly, the “CFglobal” column assesses the global impact on biodiversity, again in terms of PDF*year per kilogram of transport between the countries (XLSX)
