# Supplementary material for: What Is the Impact of Accidentally Transporting Terrestrial Alien Species? A New Life Cycle Impact Assessment Model
Source: Environ Sci Technol. 2024 Feb 8;58(7):3423–36. doi: 10.1021/acs.est.3c08500 (PMC10882960; doi:10.1021/acs.est.3c08500)
Supplement: Supplementary file 1 — es3c08500_si_001.pdf [file es3c08500_si_001.pdf]

What is the impact of accidentally transporting terrestrial alien species? A new Life Cycle Impact Assessment model

Authors

Jan Borgelt[*1], Martin Dorber[1], Charly Géron[2,3,4], Koen J. J. Kuipers[5], Mark A. J. Huijbregts[5], Francesca Verones[1]

Affiliations

1. Industrial Ecology Programme, Department of Energy and Process Engineering, Norwegian University of Science and Technology (NTNU), 7034, Trondheim, Norway

2. Biodiversity and Landscape, TERRA research centre, Gembloux Agro-Bio Tech, University of Liège, Gembloux, 5030, Belgium

3. Plants and Ecosystems, University of Antwerp, Wilrijk, 2610, Belgium

4. University of Rennes, CNRS, ECOBIO (Écosystèmes, Biodiversité, Évolution), UMR, 6553 Rennes, France

5. Department of Environmental Science, Radboud Institute for Biological and Environmental Sciences, Radboud University, 6500 GL, Nijmegen, Netherlands

*corresponding author: Jan Borgelt (jan.borgelt@ntnu.no)

Supporting Information

This file includes 4 Figures and 2 Tables on 4 pages.

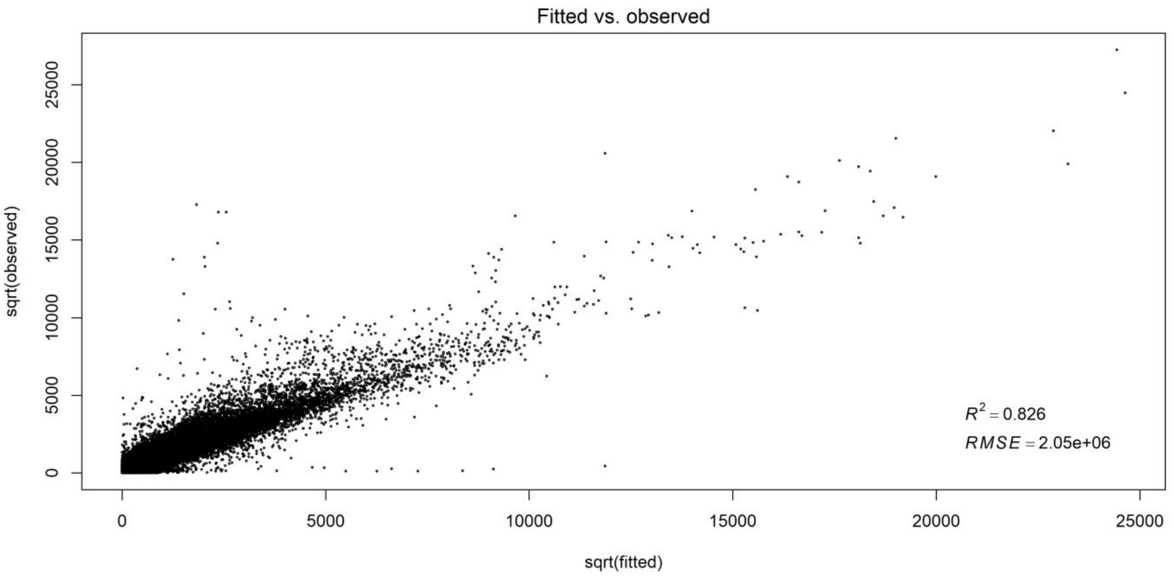

Figure S1: Predicted values of trade quantities based on trade in monetary values ($). Square root transformed fitted values versus square root transformed observed values.

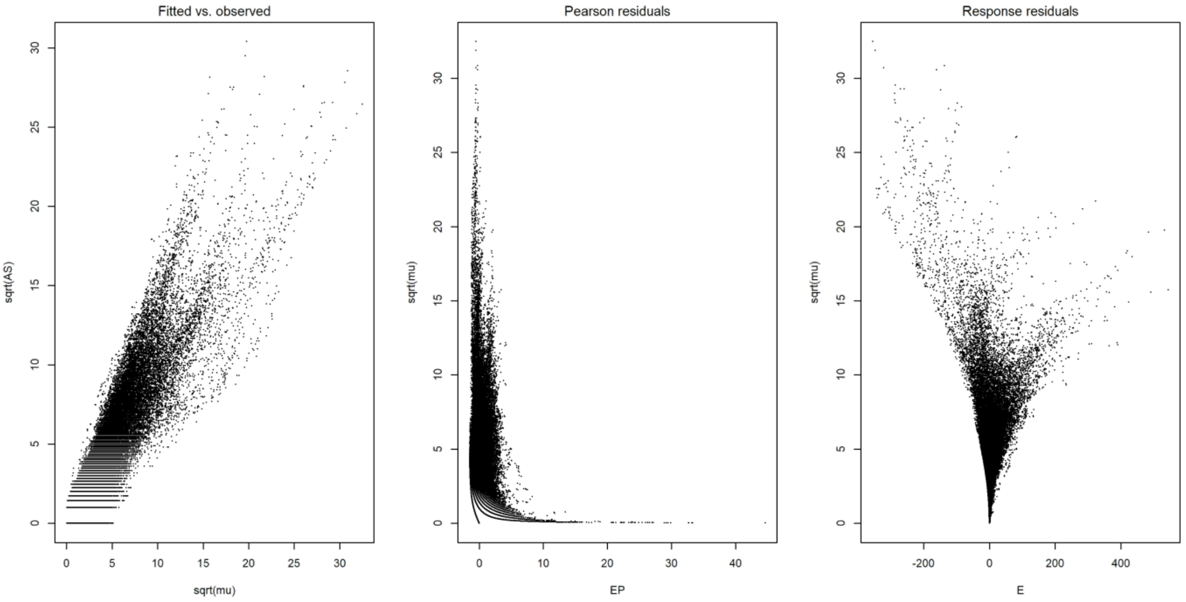

Figure S2: Square root transformed fitted values versus square root transformed observed values, Pearson residuals versus square root transformed fitted values, and raw residuals versus square root-transformed fitted values.

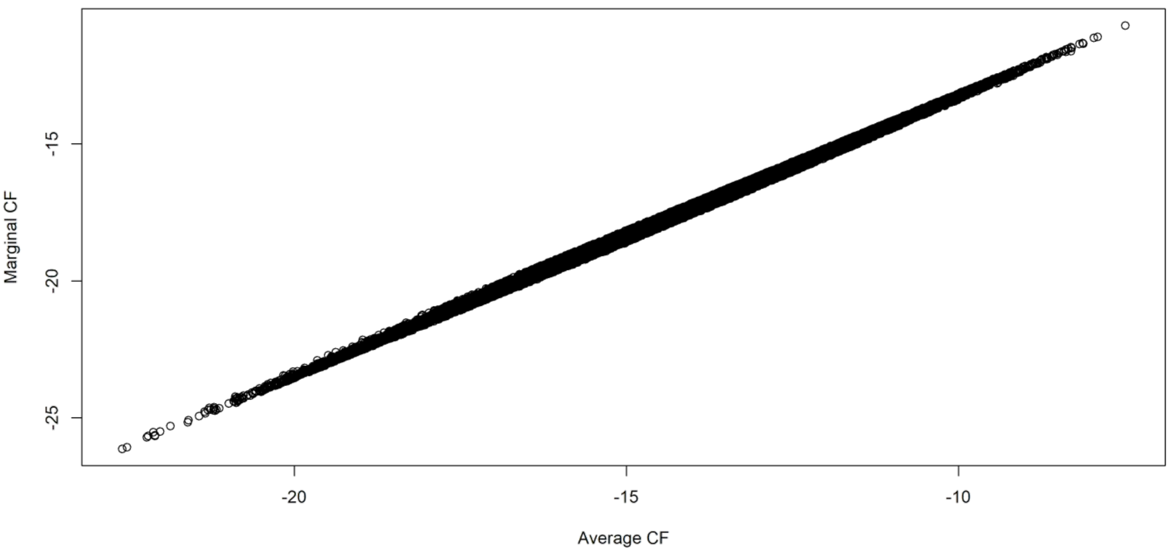

Figure S3: Log10-transformed values of country-specific marginal versus average characterization factors (CF).

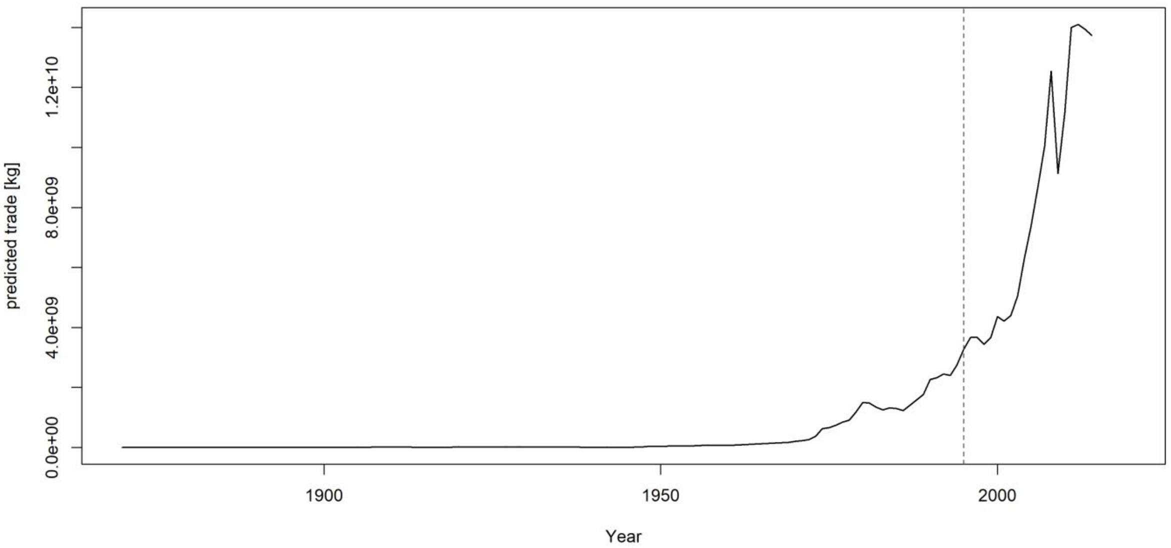

Figure S4: Predicted values of traded quantities over time. Predictions are based on trade in monetary values ($). The year 1995 delineates the timing from which trade quantities are provided in BACI[62] and is shown by dashed line.

Table S1: Possible combinations of fixed effects in generalized linear mixed models fitted by maximum likelihood (Laplace Approximation), Negative Binomial with a log link. Transported alien species ($AS_{e\rightarrow i}$) were related to transported quantity ($TR_{e\rightarrow i}$), the nearest distance ($D_{e\rightarrow i}$) between importing and exporting country, the species pool (S_e) in the exporting country, and number of native species in the importing country (S_i). Model ranking based on the corrected Akaike's Information Criterion (AICc) and the corresponding model's likelihood of being the optimal choice (W_{AICc}).

**Design**	**AICc**	W_{AICc}
$AS_{e\rightarrow i} \sim TR_{e\rightarrow i}+D_{e\rightarrow i}+S_e+S_i$ + (1|period/country$_i$) + (1|period/country$_e$)	923986.6	1
$AS_{e\rightarrow i} \sim TR_{e\rightarrow i}+D_{e\rightarrow i}+S_e$ + (1|period/country$_i$) + (1|period/country$_e$)	924049.7	0
$AS_{e\rightarrow i} \sim D_{e\rightarrow i}+S_e+S_i$ + (1|period/country$_i$) + (1|period/country$_e$)	924294.1	0
$AS_{e\rightarrow i} \sim D_{e\rightarrow i}+S_e$ + (1|period/country$_i$) + (1|period/country$_e$)	924358.7	0
$AS_{e\rightarrow i} \sim TR_{e\rightarrow i}+S_e+S_i$ + (1|period/country$_i$) + (1|period/country$_e$)	926753.1	0
$AS_{e\rightarrow i} \sim TR_{e\rightarrow i}+S_e$ + (1|period/country$_i$) + (1|period/country$_e$)	926816.5	0
$AS_{e\rightarrow i} \sim S_e+S_i$ + (1|period/country$_i$) + (1|period/country$_e$)	927462.4	0
$AS_{e\rightarrow i} \sim S_e$ + (1|period/country$_i$) + (1|period/country$_e$)	927528.1	0
$AS_{e\rightarrow i} \sim TR_{e\rightarrow i}+D_{e\rightarrow i}+S_i$ + (1|period/country$_i$) + (1|period/country$_e$)	927836.3	0
$AS_{e\rightarrow i} \sim TR_{e\rightarrow i}+D_{e\rightarrow i}$ + (1|period/country$_i$) + (1|period/country$_e$)	927899.4	0
$AS_{e\rightarrow i} \sim D_{e\rightarrow i}+S_i$ + (1|period/country$_i$) + (1|period/country$_e$)	928171.1	0
$AS_{e\rightarrow i} \sim D_{e\rightarrow i}$ + (1|period/country$_i$) + (1|period/country$_e$)	928235.8	0
$AS_{e\rightarrow i} \sim TR_{e\rightarrow i}+S_i$ + (1|period/country$_i$) + (1|period/country$_e$)	930443.6	0
$AS_{e\rightarrow i} \sim TR_{e\rightarrow i}$ + (1|period/country$_i$) + (1|period/country$_e$)	930507	0
$AS_{e\rightarrow i} \sim S_i$ + (1|period/country$_i$) + (1|period/country$_e$)	931182.8	0
$AS_{e\rightarrow i} \sim 1$ + (1|period/country$_i$) + (1|period/country$_e$)	931248.5	0

Table S2: Statistics of the best fitted generalized linear mixed model by maximum likelihood (Laplace Approximation), Negative Binomial (2.4753) with a log link, including Chi-square test statistics for the difference between the selected model and the null model ($X^2_{df,n}$), the marginal and conditional R^2 for variance explained by the fixed-effects only (R^2_m) and the entire model (R^2_c), respectively, the effect estimate (and 95% CI), and its corresponding p-value ($p_{estimate}$).

**Model**	***Fixed effect***	**Effect estimate (95% CI)**	$p_{estimate}$
**$AS_{e\rightarrow i} \sim TR_{e\rightarrow i}+D_{e\rightarrow i}+S_e+S_i$ + (1|period/country$_i$) + (1|period/country$_e$)**	TR	9.38×10^{-3} (8.33×10^{-3}, 1.04×10^{-2})	<0.001
$X^2_{4,\ 690060}$ = 7269.9 (p < 0.001)	D	-3.58×10^{-5} (-3.71×10^{-5}, -3.45×10^{-5})	<0.001
R^2_m = 0.068, R^2_c = 0.987	S_e	8.35×10^{-1} (8.12×10^{-1}, 8.58×10^{-1})	<0.001
	S_i	3.30×10^{-1} (2.08×10^{-1}, 4.53×10^{-1})	<0.001
